# Analysis of the current status and hot topics in spinal schwannoma imaging research based on bibliometrics

**DOI:** 10.3389/fneur.2024.1408716

**Published:** 2024-09-10

**Authors:** Abudunaibi Abudueryimu, Kutiluke Shoukeer, Haihong Ma

**Affiliations:** ^1^Kashi Prefecture Second People’s Hospital, Kashi, China; ^2^Department of Orthopedics, Sixth Affiliated Hospital of Xinjiang Medical University, Ürümqi, China

**Keywords:** spinal schwannoma, imaging, bibliometrics, visual analysis, MRI

## Abstract

**Objective:**

This study aims to explore the current hot topics and future research trends in spinal schwannoma imaging research, providing a reference for related studies and promoting the development of spinal schwannoma imaging.

**Methods:**

We conducted a literature search in the Web of Science database using the search terms (((TS = (Spinal schwannoma)) AND TS = (Imaging)) OR TS = (Spinal schwannoma)) AND TS = (image) to retrieve relevant articles. The collected data, including authors, keywords, journals, countries, institutions, and references, were subjected to visual analysis using the visualization software CiteSpace 6.4.2R and VOSviewer 1.6.19.

**Results:**

A total of 310 relevant articles were identified. After further screening based on time limits, inclusion, and exclusion criteria, 179 articles were included in the study, consisting of 132 original articles and 42 reviews. These articles were authored by 1,034 authors from 35 countries and 324 institutions and were published in 82 different journals. The included articles cited a total of 6,583 references from 1,314 journals.

**Conclusion:**

Although the field of spinal schwannoma imaging research is not a popular research area in the medical community, there has been an increasing international interest in this field in recent years. While China ranks high in terms of the number of published articles, there is still a gap in terms of the quality and research level compared to developed countries in Europe and America. MRI, as the gold standard for diagnosing spinal schwannomas, is expected to be a research hotspot in terms of feature analysis, enhancement characteristics, and quantitative analysis. It is also hoped that China can increase its investment in research and contribute to the field by publishing high-quality articles in the future.

## 1 Introduction

Intraspinal schwannomas are one of the most common intradural extramedullary tumors, originating from the nerve sheath cells within the spinal canal. They typically present as axial pain and neurological symptoms caused by progressive compression of the spinal cord (1, 2). With the continuous development and advancement of medical imaging technology, imaging plays a crucial role in the diagnosis, localization, and evaluation of intramedullary spinal cord tumors (3, 4). Various imaging techniques, such as magnetic resonance imaging (MRI) and computed tomography (CT) (5), provide detailed information about the tumor, including its size, shape, boundaries, internal structure, and relationship with surrounding tissues (6). Imaging studies of intramedullary spinal cord tumors not only aid in accurate diagnosis and differential diagnosis but also provide important evidence for treatment planning and surgical intervention (7). As imaging technology continues to innovate and progress, research on the imaging of intramedullary spinal cord tumors also continues to evolve (8, 9). Researchers are dedicated to exploring new imaging features, quantitative analysis methods, and the application of deep learning techniques to improve the accuracy of diagnosis and treatment outcomes for intramedullary spinal cord tumors (10, 11). Furthermore, international collaboration and communication provide a broader platform for the imaging research of intramedullary spinal cord tumors, facilitating further advancements in this field. However, despite the abundance of literature on imaging studies of intramedullary spinal cord tumors, there is currently no systematic review of the research directions and trends. Therefore, this study selected literature published in the Web of Science database to conduct an in-depth analysis of the current status and development trends in the field of imaging research on intramedullary spinal cord tumors using bibliometric methods. We aim to explore, analyze, and construct the core structure, developmental history, cutting-edge areas, and overall knowledge framework of this field, visualizing the correlations between them. It is hoped that this article will provide valuable information and insights to the medical community, promoting further progress in the imaging research and clinical applications of intramedullary spinal cord tumors.

## 2 Data and methods

### 2.1 Data collection database time limit

#### 2.1.1 Retrieval

##### 2.1.1.1 Search strategy

Open the advanced retrieval in the Web of Science (WoS) page, select the Web of Science core collection, and the retrieval strategy is shown in Table 1. Select keywords ((TS = (Spinal schwannoma)) AND TS = (Imaging)) ORTS = (Spinal schwannoma) AND TS = (image) to search the target literature.

**Table 1:** Search strategy for the Web of Science database.

	Retrieval type
#1	Subject words: Spinal schwannoma
#2	Subject words: Imaging
#3	Subject words: image
#4	#1 AND #2
#5	#1 AND #3
#6	#4 OR #5

### 2.2 Inclusion and exclusion criteria

#### 2.2.1 Inclusion criteria

① Literature on imaging studies of intraspinal schwannoma published from 2014 to 2023; ②, review; ③ in English.

#### 2.2.2 Exclusion criteria

① No relevance in this study; ② duplicate papers; ③ non-English language literature; ④ conference papers, abstract, translation, dissertation, dissertation, newspaper, lecture, news, etc.

### 2.3 Analysis of the data

Bibliometrics is a method used to analyze the production and status of publications in a specific research field from both quantitative and qualitative perspectives (12, 13). By utilizing bibliometric software like CiteSpace 6.4.2R and VOSviewer 1.6.19, we can visually analyze collected data, including authors, keywords, journals, countries, institutions, and references. VOSviewer 1.6.19 (Visualizing Scientific Landscapes) is a bibliometric analysis software commonly employed to construct collaboration, citation, and co-occurrence networks by extracting key information from numerous publications (14). In the maps generated by VOSviewer, the size and color of nodes represent the quantity and category of these items, while the thickness of the links reflects the strength of collaboration or association between items. CiteSpace 6.4.2R, developed by Professor Chaomei Chen, is another software used for bibliometric analysis and visualization. In this study, we utilized these two software tools to create visual maps and analyze the current hotspots and future trends in the field of intramedullary spinal cord tumor imaging research, taking into account factors such as publication volume, countries, institutions, journals, authors, keywords, and burst terms (15, 16).

## 3 Results

**Figure 1:**
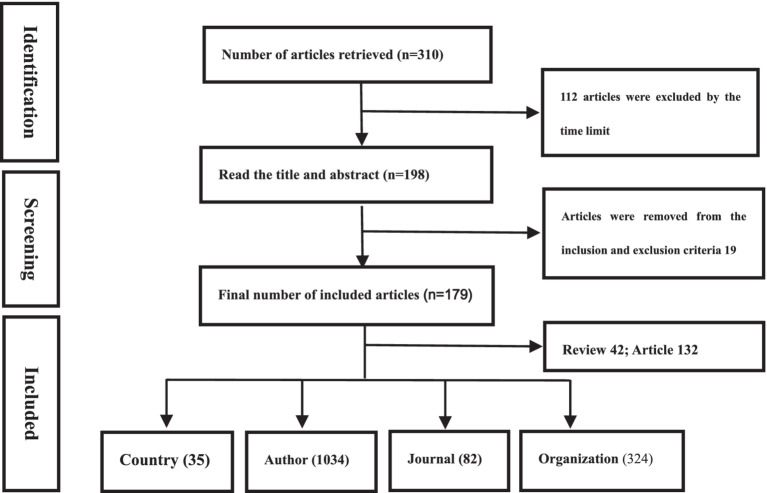
Flow chart of literature search and screening.

There are a total of 310 relevant articles in the Web of Science database. Further screening was conducted based on time limits, inclusion, and exclusion criteria (see Figure 1), resulting in a final inclusion of 179 articles, including 132 original research papers and 42 review articles. These publications were authored by 1,034 authors from 35 countries and 324 institutions, and they were published in 82 different journals. The articles cited a total of 6,583 references from 1,314 journals.

### 3.1 Global research status in the field of imaging studies for intraspinal schwannoma

**Figure 2:**
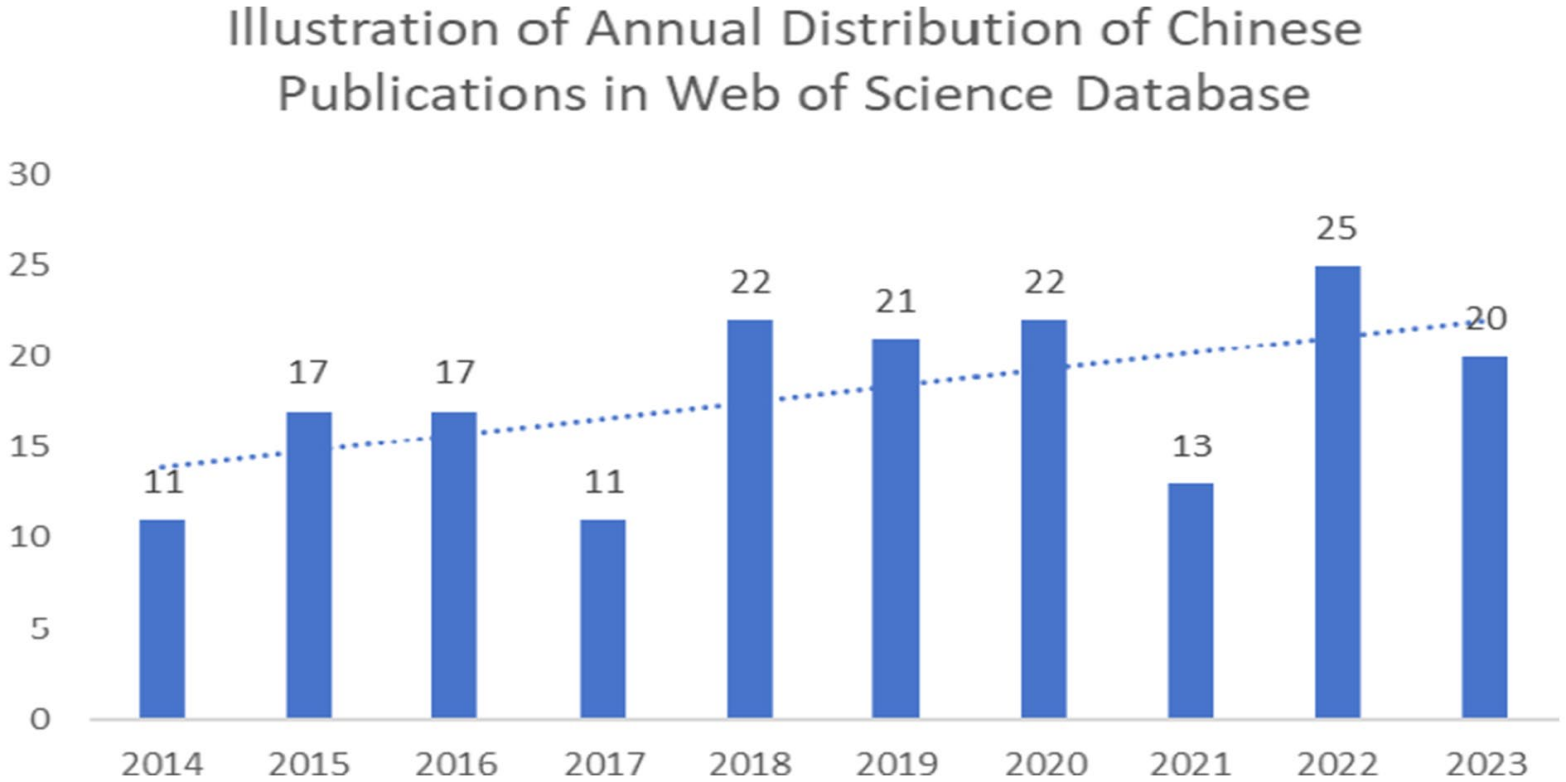
Imaging of spinal schwannoma was published in the literature.

According to the data shown in Figure 2, we can observe that the publication volume of intramedullary spinal cord tumor imaging research articles has exhibited some fluctuations over the past decade. There were relatively high publication volumes during the periods of 2015–2016 and 2020–2022, while in other years, the publication volume remained relatively stable or slightly decreased. Specifically, from 2014 to 2017, the publication volume initially increased from 11 articles to 17 articles and then dropped back to 11 articles. However, in 2018, the publication volume significantly increased to 22 articles, representing a 100% growth compared to 2017. In 2022, there was another significant increase, reaching a peak of 25 articles, the highest value in the past decade. Overall, although the publication volume fluctuated each year, there is a general increasing trend, which may reflect the growing importance and activity in research within this field.

### 3.2 National analysis of the imaging field of intraspinal schwannoma

**Figure 3:**
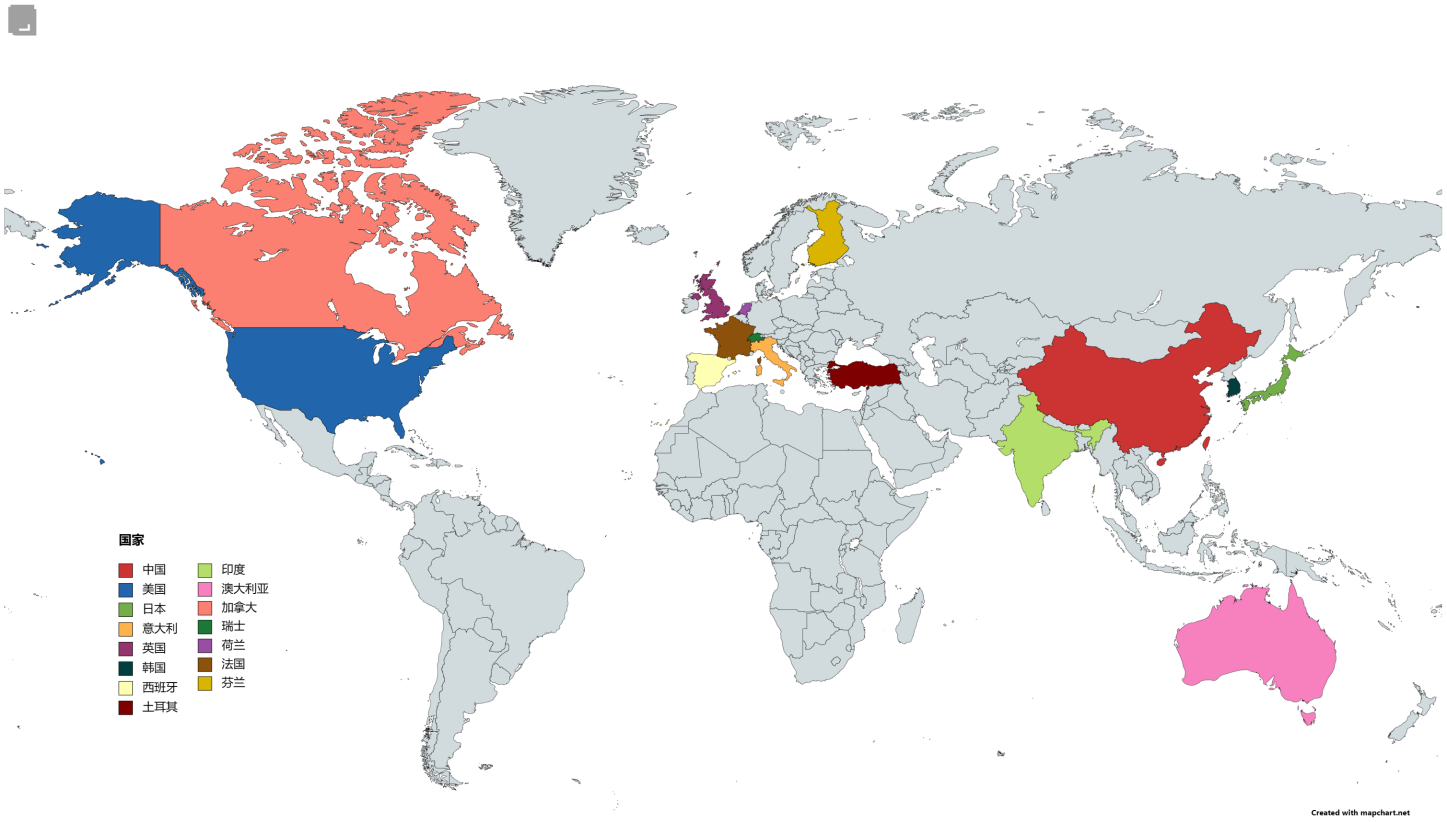
National map of the field of imaging of spinal schwannoma.

From the perspective of country distribution, Figure 3 displays the distribution of published countries in global intramedullary spinal cord tumor imaging research literature. Observing the chart, it can be noted that developed countries dominate the publication of related literature, particularly with a significant number of countries in the European region compared to other continents. This is associated with the presence of numerous developed countries in Europe. Table 2 lists the top 10 countries with the highest number of published literature globally. China has the highest publication volume in the field of intramedullary spinal cord tumor imaging research, with a total of 58 publications, ranking first. The United States and Japan rank second and third, with 30 and 29 publications, respectively. In terms of citation count, the United States has the highest citation count, reaching 340, ranking first. The United Kingdom and Japan rank second and third, with citation counts of 134 and 175, respectively. In terms of average citations per article, the United Kingdom performs remarkably well, with an average of 13.4 citations per article, ranking first. The United States and Spain rank second and third, with average citations per article of 11.3 and 11.2, respectively.

**Table 2:** Top 10 countries in the field of imaging of spinal schwannoma.

No.	Countries	Articles	Citation	Average citations
1	China	58	248	4.3
2	USA	30	340	11.3
3	Japan	29	175	6.03
4	Italy	12	56	4.6
5	U.K.	10	134	13.4
6	Korea	10	57	5.7
7	Germany	7	63	9.0
8	Spain	6	67	11.2
9	Turkey	6	6	1.0
10	India	5	12	2.4

### 3.3 National network analysis

**Figure 4:**
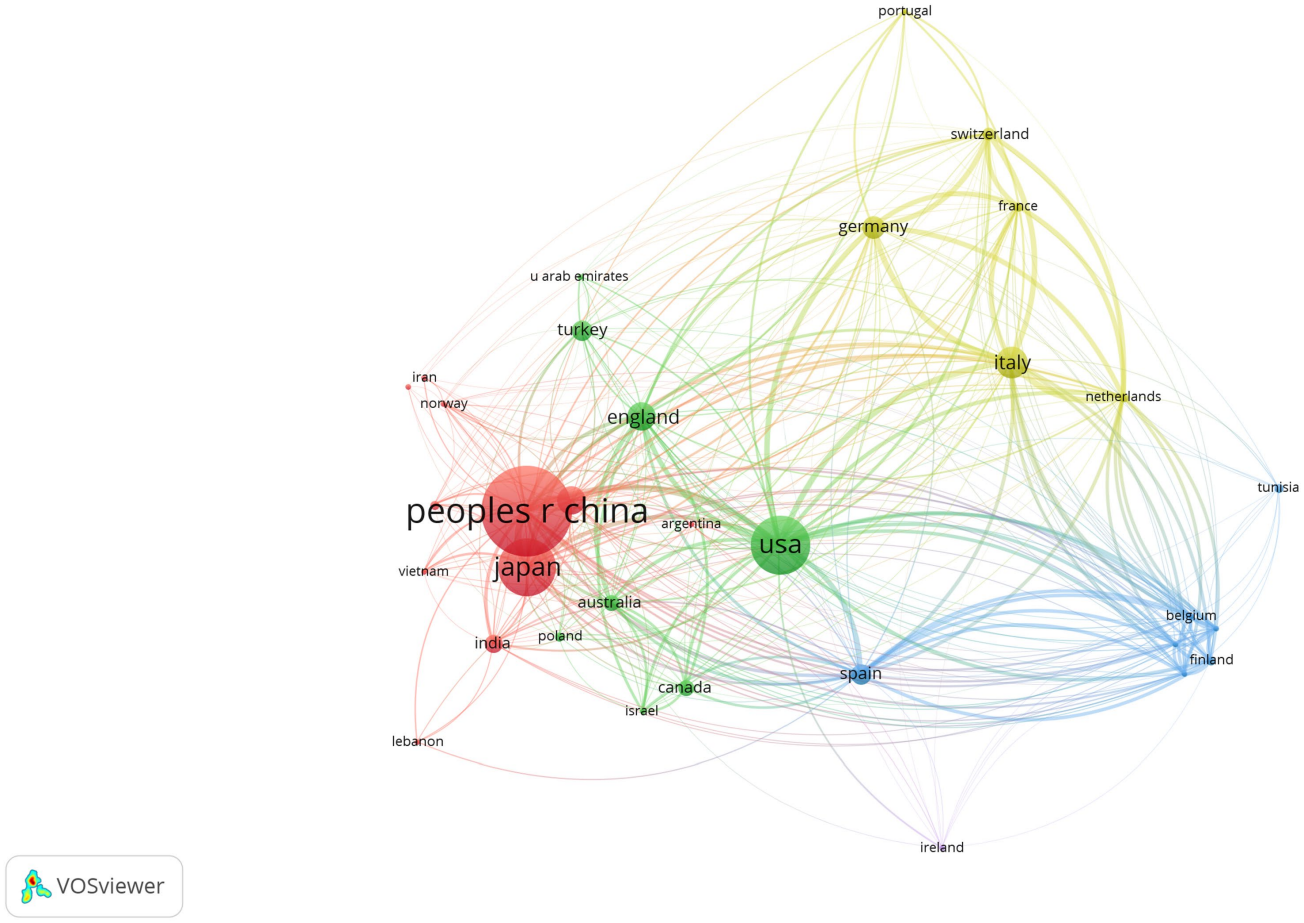
National network analysis map of the imaging field of spinal schwannoma.

The country collaboration network analysis graph (Figure 4) generated by VOSviewer 1.6.19 illustrates the collaboration network in the field of intramedullary spinal cord tumor imaging research, involving 35 countries. In this network, China, the United States, and Japan are considered the three major powerhouses in this field, with link strengths of 1,835, 2,703, and 1,164, respectively. The collaboration network between China and Asian countries such as Japan and South Korea is relatively close, while the United States collaborates more frequently with English-speaking countries like the United Kingdom, Canada, and Australia. It is worth noting that the collaboration among European countries is relatively even and close. This indicates that Europe has a well-developed research collaboration network in this field, with countries collectively driving scientific progress.

### 3.4 Study authors and institutions of intraspinal schwannoma imaging

**Figure 5:**
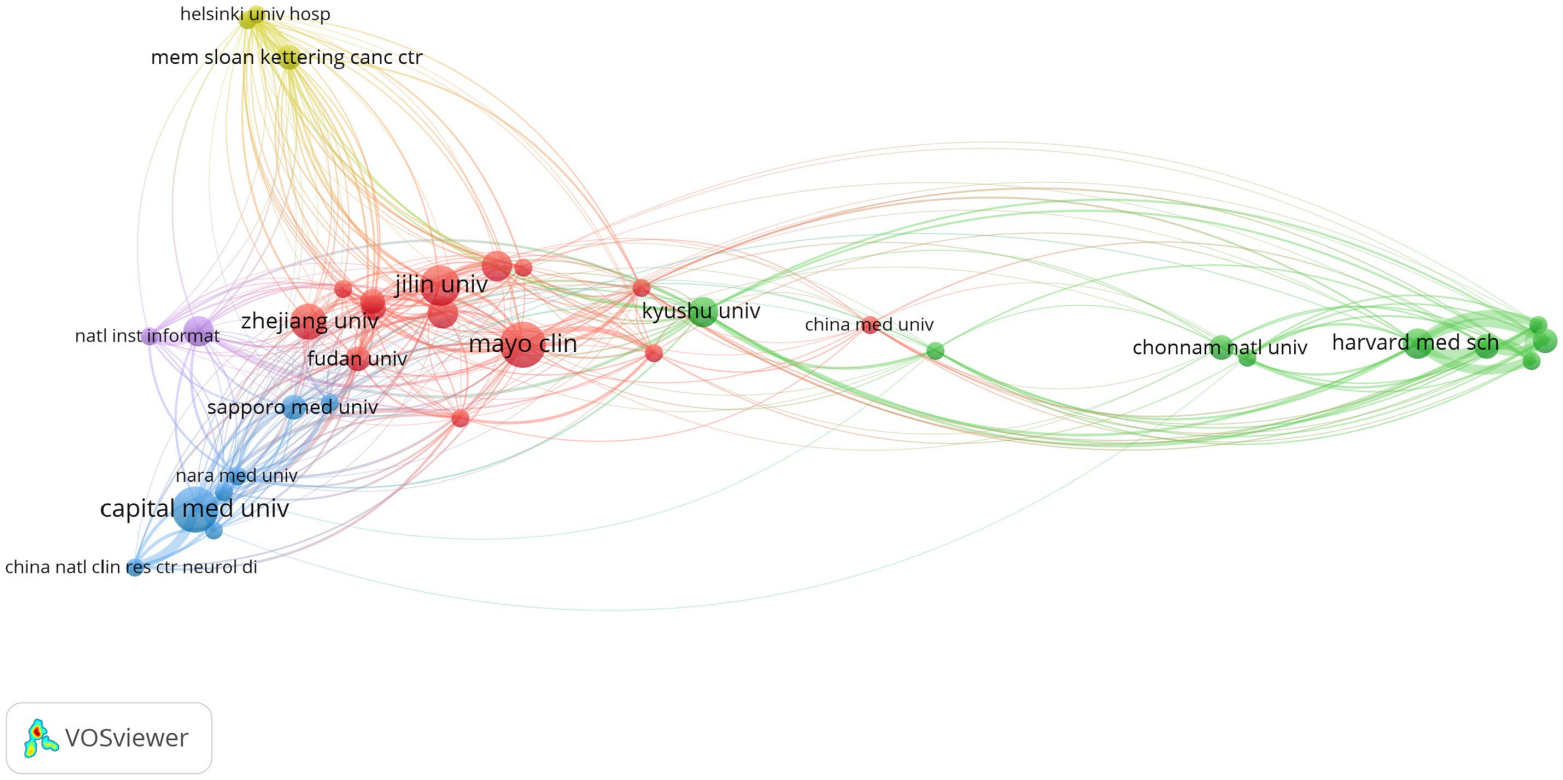
Institutional network diagram in the field of spinal schwannoma imaging research.

Analyzing the author and institution data (Table 3), it is found that the top five authors in terms of publication volume are all from Japan. Shiro Imagama ranks first with 4 publications, while Kei Ando, Naoki Ishiguro, and Kazuyoshi Kobayashi have 3 publications each. Their average citations per article are all 7.6, which is the highest. In terms of institutions (Table 4), Mayo Clinic and Capital Medical University both have 7 publications. However, Mayo Clinic has a higher citation count (83) and average citations per article (11.8) compared to Capital Medical University (49 citations and 7.0 average citations per article). This indicates that the Mayo Clinic has a greater research influence in this field. Jilin University ranks third with 6 publications, but its average citations per article are 4.0. This reflects that although Jilin University has a higher number of articles, the quality of the articles still needs improvement. Harvard University, despite having only 4 publications, has a high citation count of 130 and an average of 32.5 citations per article, which is the highest among all institutions. This indicates that Harvard University’s research in this field has a high impact and recognition. By running VOSviewer, the institution network graph (Figure 5) was obtained, with a threshold set at 2, resulting in 35 nodes and 334 connections. The 35 institutions form 5 clusters.

**Table 3:** Top 5 authors in the field of imaging of spinal schwannoma.

No.	Author	Articles	Citations	Average citations
1	Shiro Imagama	4	23	5.7
2	Kei Ando	3	23	7.6
3	Naoki Ishiguro	3	23	7.6
4	Kazuyoshi Kobayashi	3	23	7.6
5	Satoshi Tanaka	3	16	5.3

**Table 4:** Top 10 institutions in the field of imaging studies of spinal schwannoma.

No.	Institution	Articles	Citation	Average citations
1	Mayo Clinic	7	83	11.8
2	Capital Medical University	7	49	7.0
3	Jilin University	6	24	4.0
4	Harvard University	4	130	32.5
5	Kyushu University	4	31	7.75
6	University of Manchester	3	89	29.6
7	University of Toronto	3	73	24.3
8	Seoul University	3	35	11.7
9	The Seoul National University Hospital	3	35	11.7
10	Fudan University	3	10	3.3

### 3.5 Related journals in the field of imaging research of intraspinal schwannoma

**Figure 6:**
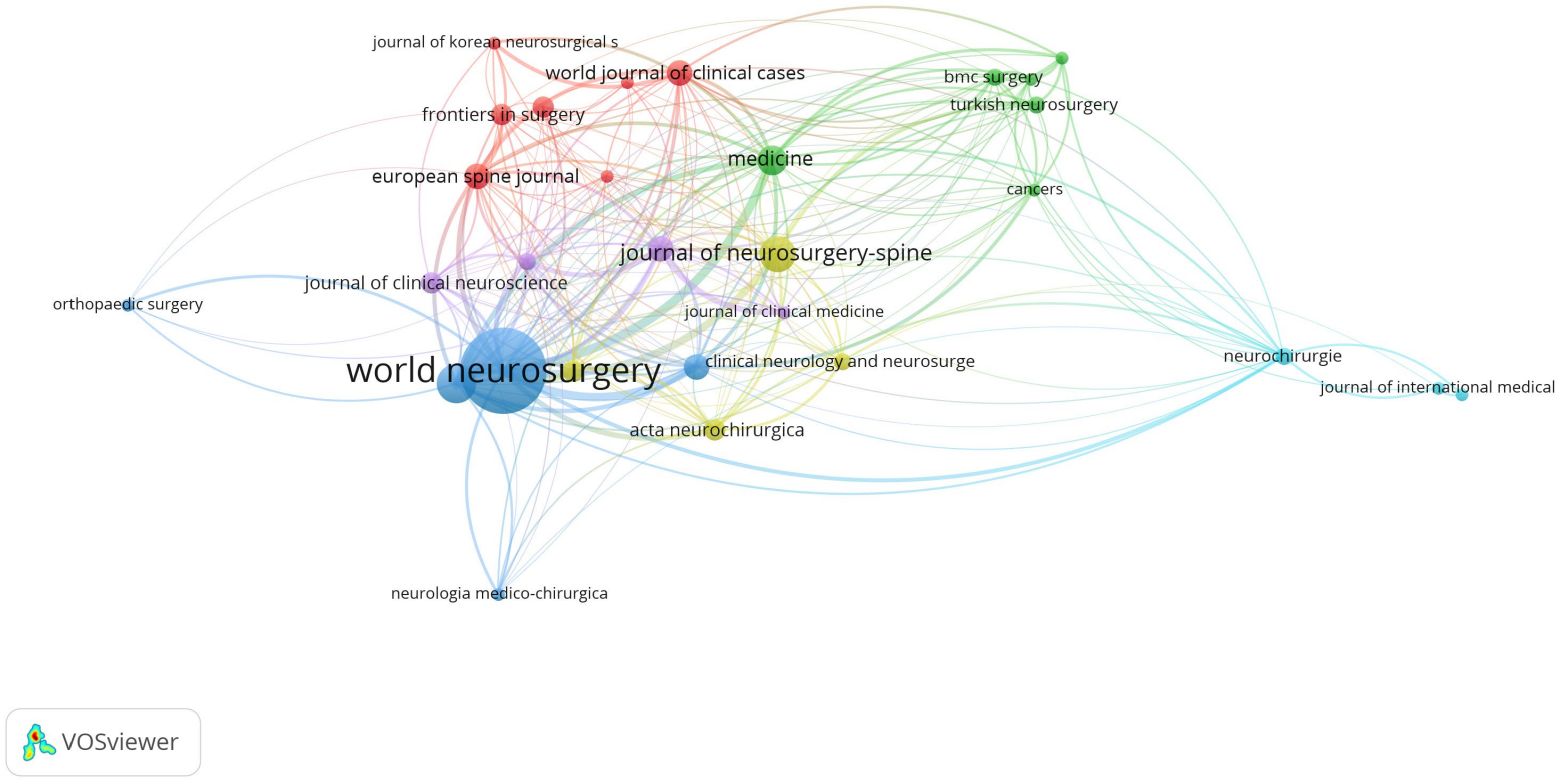
Network diagram of journals in the field of spinal schwannoma imaging research.

A total of 82 journals have published literature related to intramedullary spinal cord tumor imaging research. Among them, the journal “World Neurosurgery” has the highest number of publications (26) and also ranks first in terms of citation count (113). Although the journal “Spine” has a relatively low number of publications, it has the highest average citations per article (11.8). The journals “Journal of Neurosurgery-Spine,” “Neurosurgery,” “European Spine Journal,” “Spine,” and “Acta Neurochirurgica” are all classified as JCR Q2 journals and have relatively high impact factors, with values of 3.6, 4.6, 3.1, 3.0, and 2.2, respectively. By setting a threshold of 2 in VOSviewer, the journal network graph (Figure 6) was obtained. Twenty-nine journals were included in the graph, forming 29 nodes and 227 connections, which were divided into 6 different clusters (see Table 5).

**Table 5:** Top 10 journals on imaging studies of spinal schwannoma.

No.	Journal	Articles	Citation	Average citations	JCR (2023)	IF (2023)
1	World Neurosurgery	26	113	4.3	3	2.1+
2	British Journal of Neurosurgery	9	20	2.2	4	1.5+
3	Journal of Neurosurgery-Spine	8	84	10.5	2	3.6
4	Medicine	6	13	2.1	4	1.6
5	Neurosurgery	5	45	9.0	2	4.6
6	European Spine Journal	5	52	10.4	2	3.1
7	Spine	5	59	11.8	2	3.0
8	World Journal of Clinical Cases	5	6	1.2	4	1.3
9	Oncology Letters	4	16	4.0	3	2.9
10	Acta Neurochirurgica	4	52	13.0	2	2.2

### 3.6 Keywords in the field of lumbar spinal stenosis and treatment

**Figure 7:**
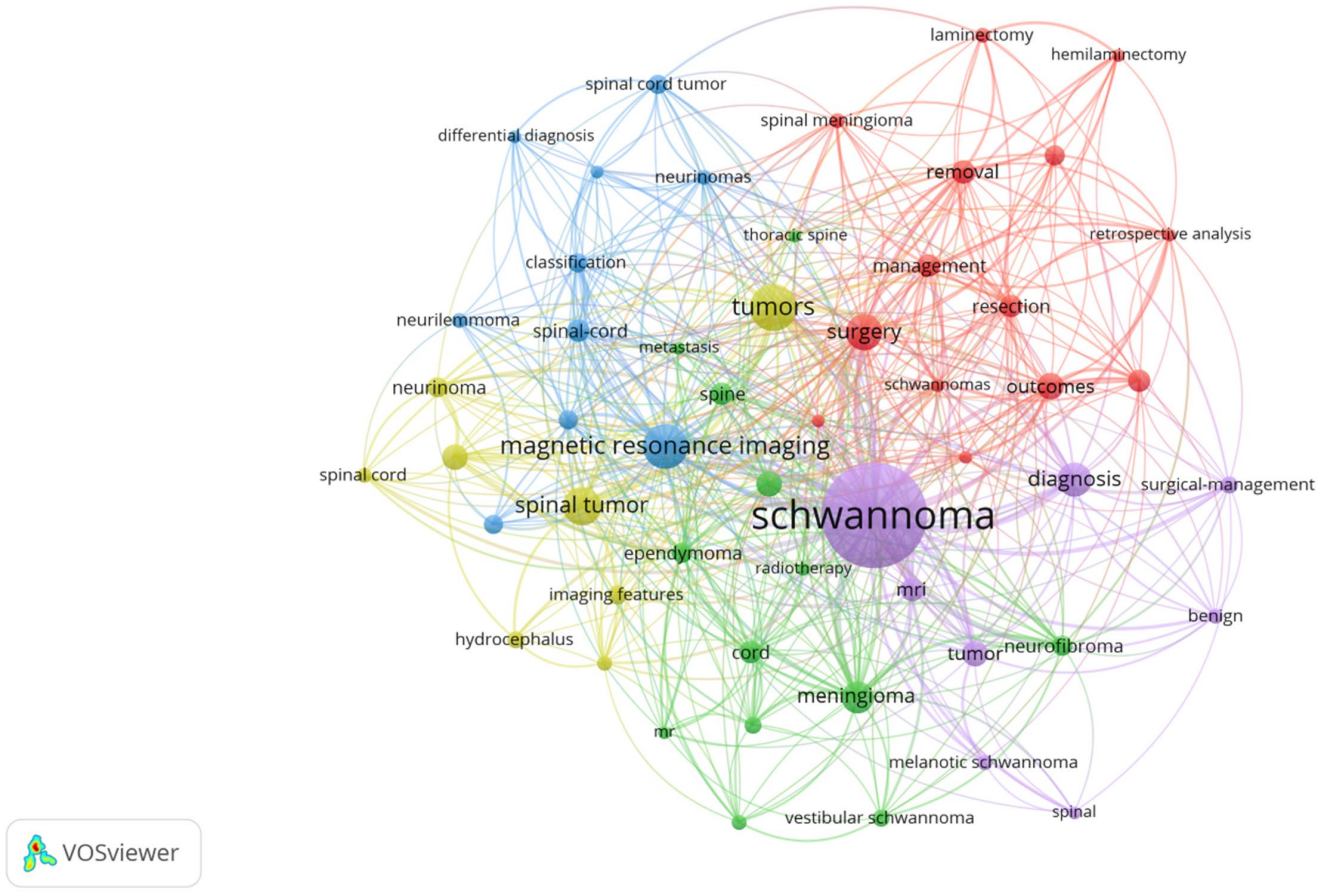
Clustering of keywords in the field of spinal schwannoma imaging research.

**Figure 8:**
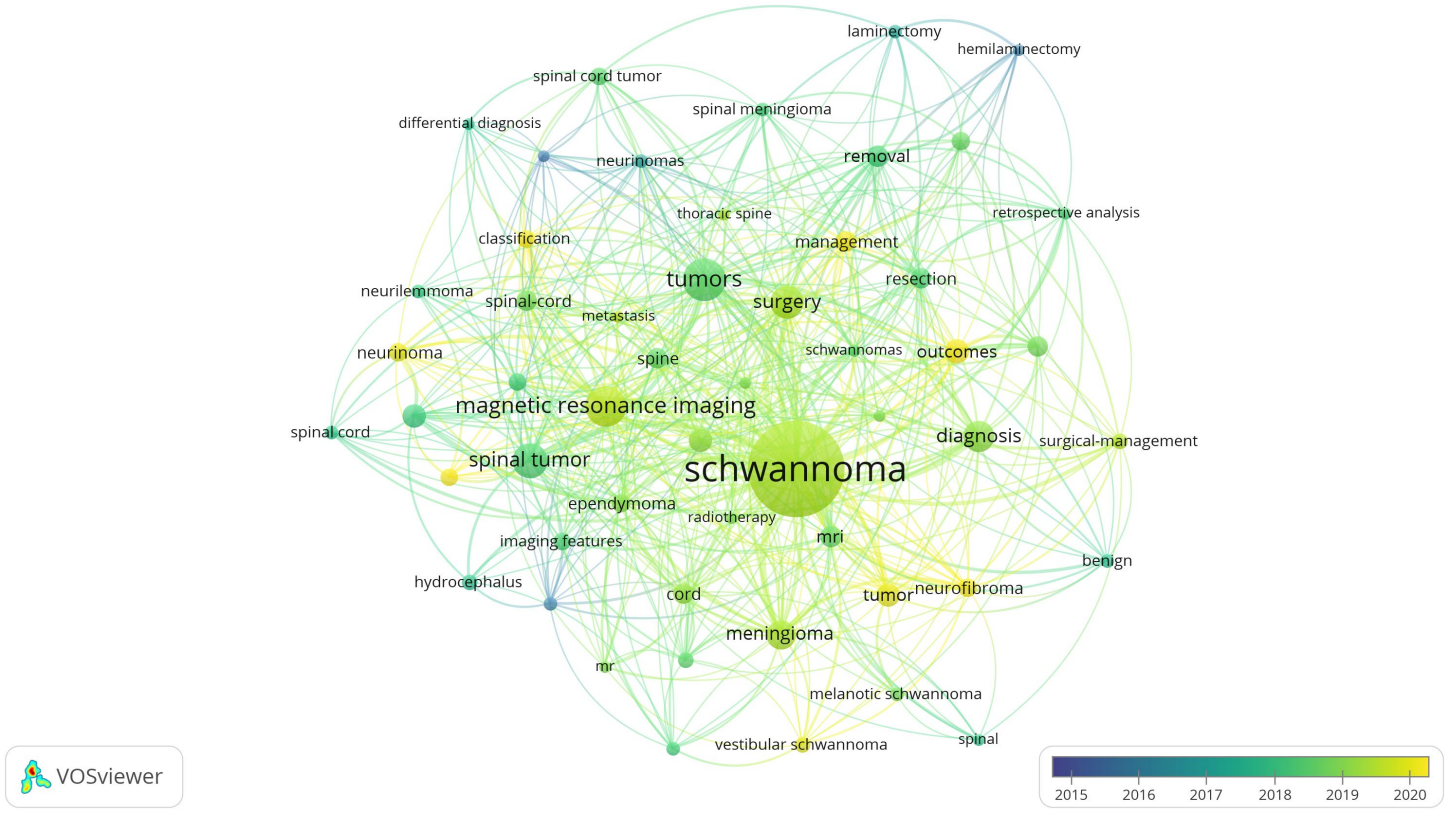
Keywords co-occurrence cluster map in the imaging field of spinal schwannoma (time superposition).

Keywords play an important role in an article as they not only summarize and reflect the core content of the article but also help readers understand the research field and related concepts, thereby enhancing their understanding of the article. Table 6 lists the top 10 keywords with the highest frequency in the literature related to intramedullary spinal cord tumor imaging research. Among them, “Schwannoma” is the most frequently appearing keyword, with a total of 88 occurrences. “Tumors” and “Magnetic resonance imaging” rank second and third with frequencies of 27 and 26, respectively. By setting a threshold of 5 in VOSviewer, a keyword network graph was generated (Figure 7). A total of 53 keywords formed 53 nodes and 525 connections, divided into 5 different clusters. It is worth noting that “Schwannoma,” “Tumors,” “Magnetic resonance imaging,” and “Surgery” became the core keywords in their respective clusters. Figure 8 illustrates the association between keyword frequency and time, where nodes closer to yellow indicate keywords that have been prominent in recent research. It can be observed that most keywords have appeared frequently in the literature from 2018 to 2023.

**Table 6:** Top 10 keywords in the field of imaging of intraspinal schwannoma.

No.	Keywords	Frequency of occurrence	Total connection strength
1	Schwannoma	82	88
2	Tumors	27	44
3	Magnetic resonance imaging	26	37
4	Spinal tumor	21	32
5	Surgery	19	26
6	Diagnosis	18	32
7	Meningioma	16	29
8	Outcomes	13	17
9	Features	12	19
10	Cauda-equina	12	18

### 3.7 Outbreak words in the field of intraspinal schwannoma

**Figure 9:**
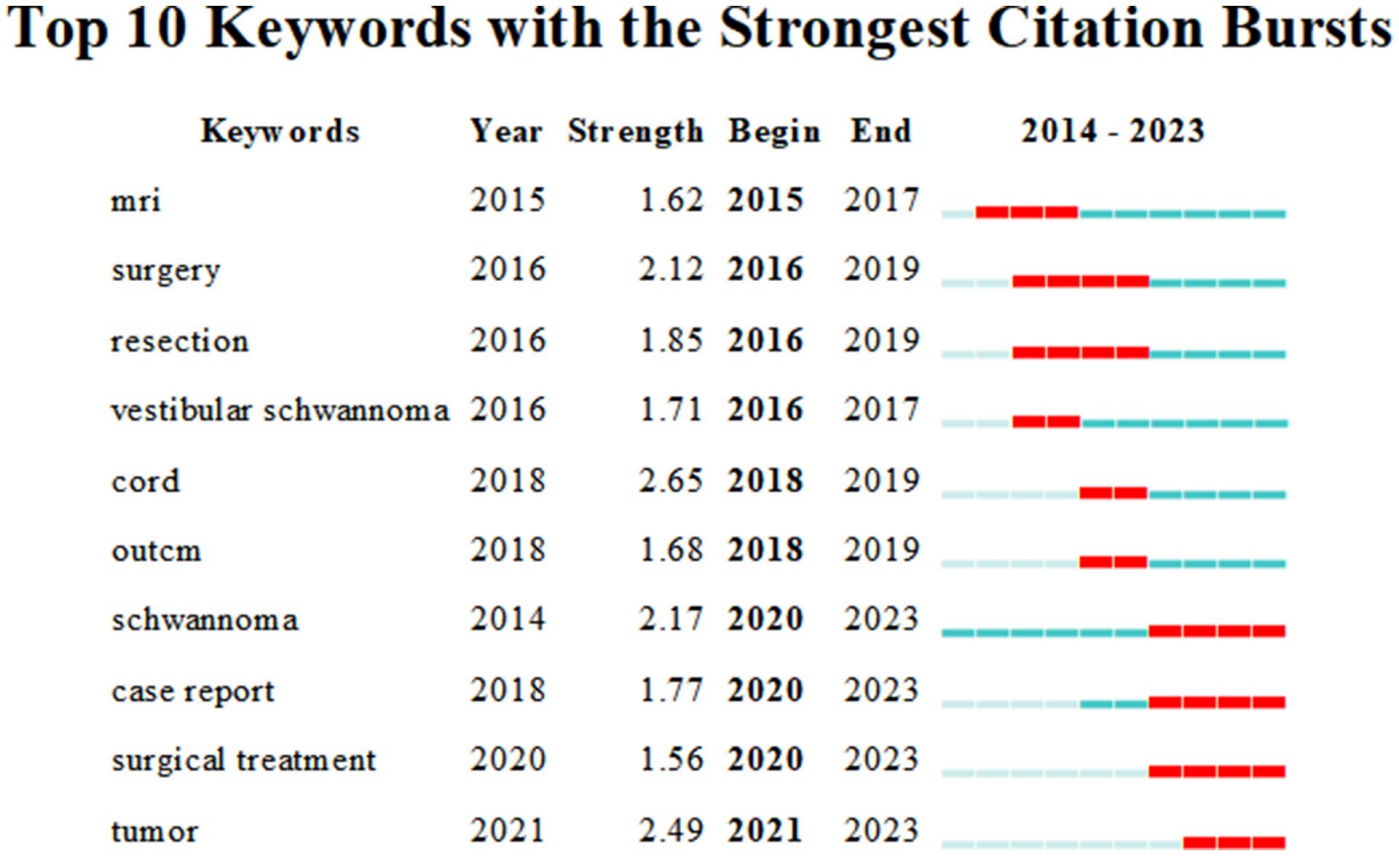
Atlas of top 10 outbreak words in the field of spinal schwannoma.

Burst terms refer to the phenomenon where a specific keyword experiences a significant increase in frequency within a particular period. Figure 9 reflects the top 10 burst terms in the field of intramedullary spinal cord tumor imaging research over the past decade. Among them, the keyword “Cord” has the strongest burst intensity with a value of 2.65. The keywords “surgery,” “resection,” “schwannoma,” and “case report” have the longest burst duration, consistently appearing at a high frequency for 3 years. Among them, the keyword “tumor” had its first burst appearance in 2021, with a burst intensity of 2.49.

## 4 Discussion

### 4.1 Bibliometry

Web of Science, developed and maintained by Clarivate Analytics, is a comprehensive academic resource platform widely used across various disciplines. It features over 8,700 high-quality academic journals, conference proceedings, patents, and other literature sources (17). Web of Science offers robust search and filtering capabilities, enabling users to efficiently locate the academic literature they need. Additionally, it provides citation indexing, allowing researchers to track the citations received by specific articles, which facilitates an understanding of their academic impact and research trends within particular fields. Overall, the Web of Science serves as an essential resource for academic researchers, scientists, and students, supporting them in conducting literature reviews, exploring new research areas, and staying informed about the latest academic developments.

### 4.2 Status of global research in the field of schwannoma

From the perspective of publication volume, the average number of publications per year in this field is 17.9, which is relatively low compared to other fields. However, there has been a noticeable upward trend in the number of publications in this field over the past decade, indicating that it is gradually gaining attention and recognition in the medical community. From a country perspective, China has almost the same publication volume (58) as the second and third-ranked countries, the United States (30) and Japan (29). This suggests that China has invested significant resources and efforts in research related to intramedullary spinal cord tumor imaging. Citation count is a reliable indicator of article quality. Similarly, the average citations per article from a country or institution can be used to evaluate the research quality and scientific level in that field. Among them, countries such as the United States and the United Kingdom, as well as institutions like Mayo Clinic and the University of Manchester, have the highest average citations per article, indicating their research level and quality are at a global top level. In contrast, although China has the highest number of articles, there is still a noticeable gap in terms of quality compared to developed countries in Europe and North America. In terms of authors, Japanese scholars have made significant contributions to this field. For example, in a retrospective study conducted in 2017, Kobayashi et al. (18) concluded that contrast-enhanced T1-weighted magnetic resonance imaging (MRI) is useful for predicting the proliferative activity and growth of intramedullary spinal cord tumors. These features are related to tumor enlargement and adhesion. The presence of an uneven pattern on contrast-enhanced T1-weighted images indicates tumor enlargement and adhesion. This pattern reflects the preoperative and postoperative motion status and recovery. In another study in 2023, Professor Shiro Imagama and his team developed an automated diagnostic system that uses deep learning with the You Only Look Once (YOLO) version 3 software, paired with MRI data, to accurately locate intramedullary spinal cord tumors. This system can detect incidental schwannomas on MRI scout images, reducing the workload of radiologists (19).

### 4.3 Future trends in the field of schwannoma

Through the analysis of keywords and burst terms, we can gain insights into the research trends and emerging topics in a specific field. Schwannoma and magnetic resonance imaging (MRI) are undoubtedly the primary research focuses in the field of intramedullary spinal cord tumor imaging. MRI, a non-invasive medical imaging technique, plays a crucial role in generating detailed images of human tissue structures (20). It not only aids in accurate tumor localization but also provides valuable information about tumor shape, boundaries, internal structure, and relationship with surrounding tissues (21). Distinguishing intramedullary spinal cord tumors from meningiomas has always been a challenging task in radiology (22). In a study evaluating the effectiveness of MRI in 764 cases of brain tumors, the sensitivity of MRI for schwannomas ranged from 90.7 to 92.6%, while for meningiomas, it ranged from 88.4 to 95.7% (23). Due to the similarity in imaging features between intramedullary spinal cord tumors and meningiomas, approximately 25% of schwannomas and meningiomas are difficult to differentiate in diagnosis (24, 25). In another study, researchers confirmed that the signal intensity ratio between intramedullary tumors and fat on T2-weighted images can accurately differentiate schwannomas from meningiomas (26). The preoperative identification of filum terminale ependymomas (FTE) and schwannomas poses significant challenges but is vital for the formulation of surgical plans and the assessment of prognoses. In a retrospective analysis, Gu et al. (27) identified that key elements include contrast-enhanced magnetic resonance imaging (MRI), convolutional neural networks (CNNs), filum terminale ependymoma, and schwannoma. 8F-FDG PET/CT is a sophisticated medical imaging technique that integrates positron emission tomography (PET) and computed tomography (CT) and is extensively utilized in fields such as oncology, cardiology, and neuroscience. In a case report by Gültekin et al. (28), the team successfully diagnosed a patient with multiple sclerosis and liver metastasis using 8F-FDG PET/CT, with subsequent biopsy results corroborating their diagnosis. Additionally, numerous other case reports have demonstrated that as technology continues to evolve, the role of PET-CT in the management of nerve sheath tumors is becoming increasingly significant. The synergistic application of this technology enhances the diagnostic accuracy for nerve sheath tumors, aiding physicians in developing optimal treatment plans and ultimately improving patient outcomes. A populous country, China has a high incidence of intramedullary spinal cord tumors among spinal tumors (29). Imaging plays a crucial role in the clinical diagnosis of intramedullary spinal cord tumors. Surgery is the main treatment modality for intramedullary spinal cord tumors (2), and imaging plays a key guiding role in surgical treatment, helping surgeons determine the surgical approach, extent of resection, and protection of surrounding neural structures (30). Additionally, imaging can be used for postoperative follow-up and evaluation of treatment outcomes, monitoring tumor recurrence or progression (31). The imaging diagnosis of intramedullary spinal cord tumors remains a core issue in this field and is one of the main directions for future development. Therefore, improving the level of imaging diagnosis for intramedullary spinal cord tumors is an important task for healthcare professionals in China. Only through the combination of imaging and scientific research efforts can solid theoretical foundations be provided for clinical treatment, improving the level of care and enhancing the quality of patient prognosis.

### 4.4 Lack of the study

This study only includes relevant literature from the Web of Science Core database. Although this database has extensive coverage and includes a wide range of journals, there may still be some data omissions. Additionally, this study only includes English-language literature and may not capture high-quality non-English literature, which could introduce selection bias. Furthermore, the Web of Science Core database is continuously updated, so the analysis results are time-limited. However, the existing research still provides valuable insights and guidance for our research direction and design.

## 5 Conclusion

This article leverages bibliometric analysis of pertinent literature from the Web of Science core database to delve into the relationship between imaging studies and the clinical management of spinal intradural schwannomas. Through a comprehensive statistical analysis of an extensive body of literature, this study uncovers prevailing research trends, identifies hot topics, and maps out the knowledge landscape, thus significantly enriching the traditional literature review approach. Employing an interdisciplinary strategy, it promotes dialogue and cooperation among various fields, thereby broadening and deepening the scope of research into spinal intradural schwannoma imaging. Bibliometrics offers a swift and efficient means to process and analyze vast amounts of literature data, equipping researchers with timely and precise insights into the current state and directions of research. This method boosts research efficiency, minimizes repetitive studies, and fosters the rapid development and innovation of knowledge. Although spinal intradural schwannomas have not traditionally been a focal point in medical research, heightened international interest and investment have brought increased attention to this area. Nonetheless, there is a pressing need for enhancement in research quality, addressing prevailing issues such as the overall low quality of studies and a scarcity of mechanistic research. MRI remains the definitive diagnostic tool for spinal intradural schwannomas, with future research expected to concentrate on feature analysis, enhancement studies, and quantitative assessments, marking them as the next frontiers in research. Progress in these domains promises to raise the bar for diagnostic and therapeutic approaches to spinal intradural schwannomas, ultimately improving patient care and outcomes.

## Data Availability

The original contributions presented in the study are included in the article/supplementary materials, further inquiries can be directed to the corresponding author.

## References

[1] Ram S Vivek V Shekhar R Gabbita AC Ganesh K. Giant cervicodorsal schwannoma. J Exp Ther Oncol. (2019) 13:155–8. PMID: 31881132

[2] Ferreira A Blanco CMB Trindade JVC Mattos G Joaquim A. Surgical outcome of spinal schwannoma and neurofibroma. Rev Assoc Med Bras. (2023) 69:e20230190. doi: 10.1590/1806-9282.20230190, PMID: 37729358 PMC10508894

[3] Alektoroff K Moulopoulos LA Papanagiotou P. Spinal tumors. Radiologe. (2021) 61:267–74. doi: 10.1007/s00117-021-00815-5, PMID: 33570679

[4] Garaud S Boto J Egervari K Vargas MI. Extradural spinal meningioma mimicking a schwannoma: magnetic resonance imaging findings. Can J Neurol Sci. (2022) 49:467–9. doi: 10.1017/cjn.2021.120, PMID: 34075867

[5] Nakamae T Kamei N Tamura T Maruyama T Nakao K Farid F . Differentiation of the intradural extramedullary spinal tumors, schwannomas, and meningiomas utilizing the contrast ratio as a quantitative magnetic resonance imaging method. World Neurosurg. (2024) 188:e320–5. doi: 10.1016/j.wneu.2024.05.106, PMID: 38797281

[6] Arima H Hasegawa T Togawa D Yamato Y Kobayashi S Yasuda T . Feasibility of a novel diagnostic chart of intramedullary spinal cord tumors in magnetic resonance imaging. Spinal Cord. (2014) 52:769–73. doi: 10.1038/sc.2014.127, PMID: 25091110

[7] Ottenhausen M Ntoulias G Bodhinayake I Ruppert FH Schreiber S Förschler A . Intradural spinal tumors in adults-update on management and outcome. Neurosurg Rev. (2019) 42:371–88. doi: 10.1007/s10143-018-0957-x, PMID: 29455369

[8] Minhas AS Oliver R. Magnetic resonance imaging basics. Adv Exp Med Biol. (2022) 1380:47–82. doi: 10.1007/978-3-031-03873-0_3 36306094

[9] Harisinghani MG O’Shea A Weissleder R. Advances in clinical MRI technology. Sci Transl Med. (2019) 11:eaba2591. doi: 10.1126/scitranslmed.aba2591 31852796

[10] Ahlawat S Blakeley JO Langmead S Belzberg AJ Fayad LM. Current status and recommendations for imaging in neurofibromatosis type 1, neurofibromatosis type 2, and schwannomatosis. Skeletal Radiol. (2020) 49:199–219. doi: 10.1007/s00256-019-03290-1, PMID: 31396668

[11] Morrison DR Sorace AG Hamilton E Moore LS Houson HA Udayakumar N . Predicting schwannoma growth in a tumor model using targeted imaging. Otol Neurotol. (2021) 42:e615–23. doi: 10.1097/MAO.0000000000003063, PMID: 33661237 PMC9762121

[12] Wu F Gao J Kang J Wang X Niu Q Liu J . Knowledge mapping of exosomes in autoimmune diseases: a bibliometric analysis (2002–2021). Front Immunol. (2022) 13:939433. doi: 10.3389/fimmu.2022.939433, PMID: 35935932 PMC9353180

[13] Wang B Xing D Zhu Y Dong S Zhao B. The state of exosomes research: a global visualized analysis. Biomed Res Int. (2019) 2019:1–10. doi: 10.1155/2019/1495130 PMC6470441 31073519

[14] van Eck NJ Waltman L. Software survey: VOSviewer, a computer program for bibliometric mapping. Scientometrics. (2010) 84:523–38. doi: 10.1007/s11192-009-0146-3, PMID: 20585380 PMC2883932

[15] Synnestvedt MB Chen C Holmes JH. CiteSpace II: visualization and knowledge discovery in bibliographic databases. AMIA Annu Symp Proc. (2005) 2005:724–8. 16779135 PMC1560567

[16] McGee S Sipos T Allin T Chen C Greco A Bobos P . A systematic review of the measurement properties of performance-based functional tests in patients with neck disorders. BMJ Open. (2019) 9:e031242. doi: 10.1136/bmjopen-2019-031242, PMID: 31767589 PMC6886974

[17] Falagas ME Pitsouni EI Malietzis GA Pappas G. PubMed, Scopus, Web of Science, and Google Scholar: strengths and weaknesses. FASEB J. (2008) 22:338–42. doi: 10.1096/fj.07-9492LSF, PMID: 17884971

[18] Kobayashi K Imagama S Ando K Hida T Ito K Tsushima M . Contrast MRI findings for spinal schwannoma as predictors of tumor proliferation and motor status. Spine. (2017) 42:E150–5. doi: 10.1097/BRS.0000000000001732, PMID: 27306258

[19] Ito S Nakashima H Segi N Ouchida J Oda M Yamauchi I . Automated detection and diagnosis of spinal schwannomas and meningiomas using deep learning and magnetic resonance imaging. J Clin Med. (2023) 12:5075. doi: 10.3390/jcm12155075, PMID: 37568477 PMC10419638

[20] What is an MRI scan and what can it do? Drug Ther Bull. (2011) 49:141–4. doi: 10.1136/dtb.2011.02.0073 22170411

[21] Wald LL. Ultimate MRI. J Magn Reson. (2019) 306:139–44. doi: 10.1016/j.jmr.2019.07.016, PMID: 31350164 PMC6708442

[22] Neupane S Kashyap A Paudel S Bhattarai G Kharel SK Adhikari A . A rare case of schwannomatosis with meningioma: a case report. Ann Med Surg. (2024) 86:1724–8. doi: 10.1097/MS9.0000000000001738, PMID: 38463125 PMC10923266

[23] Yan PF Yan L Zhang Z Salim A Wang L Hu TT . Accuracy of conventional MRIs for preoperative diagnosis of intracranial tumors: a retrospective cohort study of 762 cases. Int J Surg. (2016) 36:109–17. doi: 10.1016/j.ijsu.2016.10.023, PMID: 27773598

[24] Iwata E Shigematsu H Yamamoto Y Kawasaki S Tanaka M Okuda A . Preliminary algorithm for differential diagnosis between spinal meningioma and schwannoma using plain magnetic resonance imaging. J Orthop Sci. (2018) 23:408–13. doi: 10.1016/j.jos.2017.11.012, PMID: 29198491

[25] Zhai X Zhou M Chen H Tang Q Cui Z Yao Y . Differentiation between intraspinal schwannoma and meningioma by MR characteristics and clinic features. Radiol Med. (2019) 124:510–21. doi: 10.1007/s11547-019-00988-z, PMID: 30684254

[26] Takashima H Takebayashi T Yoshimoto M Onodera M Terashima Y Iesato N . Differentiating spinal intradural-extramedullary schwannoma from meningioma using MRI T_2_ weighted images. Br J Radiol. (2018) 91:20180262. doi: 10.1259/bjr.20180262, PMID: 30052467 PMC6319836

[27] Gu Z Dai W Chen J Jiang Q Lin W Wang Q . Convolutional neural network-based magnetic resonance image differentiation of filum terminale ependymomas from schwannomas. BMC Cancer. (2024) 24:350. doi: 10.1186/s12885-024-12023-0, PMID: 38504164 PMC10949807

[28] Gültekin A Aydoğmuş Ü Arifoğlu H Bir F Yaylalı O. An intrathoracic schwannoma case in 18F-FDG PET/CT scan. Hell J Nucl Med. (2020) 23:206–8. doi: 10.1967/s002449912111 32716413

[29] Ding Y Zhu T. Progress in diagnosis and treatment of multiple schwannoma. Chin J Neurosurg. (2017) 33:426–9. doi: 10.3760/cma.j.issn.1001-2346.2017.04.028

[30] Zhang E Zhang J Lang N Yuan H. Spinal cellular schwannoma: an analysis of imaging manifestation and clinicopathological findings. Eur J Radiol. (2018) 105:81–6. doi: 10.1016/j.ejrad.2018.05.025, PMID: 30017303

[31] Merhemic Z Stosic-Opincal T Thurnher MM. Neuroimaging of spinal tumors. Magn Reson Imaging Clin N Am. (2016) 24:563–79. doi: 10.1016/j.mric.2016.04.007 27417401

